# Spatio-Temporal Distribution and Stranding Records of Cetaceans Along the Romanian Black Sea

**DOI:** 10.3390/ani16213422

**Published:** 2026-10-29

**Authors:** George-Emanuel Harcotă, Andreea-Mădălina Ciucă

**Affiliations:** Marine Ecology and Biology Department, National Institute for Marine Research and Development “Grigore Antipa”, 300 Mamaia Blvd., 900581 Constanța, Romania

**Keywords:** Black Sea, cetaceans, spatio-temporal distribution, seasonal occurrence, mortality, strandings

## Abstract

This study investigates cetacean occurrence and stranding records along the Romanian Black Sea coast between 2021 and 2025, focusing on the three resident subspecies: *Tursiops truncatus ponticus*, *Delphinus delphis ponticus*, and *Phocoena phocoena relicta*. A total of 1392 live individuals were documented across 188 sighting events, with *T. t. ponticus* and *D. d. ponticus* together representing 88.3% of all recorded individuals. The monthly distribution of recorded sighting events differed significantly among subspecies (*p* < 0.001), as did the distance of recorded sightings from the coastline (*p* < 0.001). Median distances were 4.40 km for *T. t. ponticus*, 61.78 km for *D. d. ponticus*, and 30.44 km for *P. p. relicta*. During the same period, 76 stranded cetaceans were recorded along the Romanian Black Sea coast, of which 58 were identified to subspecies level. *P. p. relicta* accounted for 70.7% of identified stranded individuals, compared with 11.7% of live individuals recorded during research cruises. Stranding records were most frequent in August and May, although their monthly distribution did not differ significantly among subspecies. Although both datasets covered the same geographical region and study period, differences in observation methods and sampling effort limit direct comparisons and prevent inferences about mortality rates or population trends. Nevertheless, the observed differences in the spatial and monthly distribution of live sightings, together with the high representation of *P. p. relicta* among stranded individuals, provide complementary information to support future cetacean monitoring, stranding investigations, and conservation efforts in the Romanian Black Sea.

## Simple Summary

To support the conservation of dolphins and porpoises in the Black Sea, we analyzed observations collected in Romanian waters and along the coastline between 2021 and 2025. Protecting these mammals requires data on where and when live individuals are observed and dead ones are discovered. The 188 reports accounted for a total of 1392 live animals, approximately 88.3% of which were bottlenose dolphins and common dolphins. In general, bottlenose dolphins were observed close to the shore, porpoises at intermediate distances, and common dolphins further offshore. The timing of these observations varied, particularly for the two dolphin species, with peaks in August and September. Of the 76 dead animals recorded, 58 were identified to the species level; approximately 71% of these were porpoises. Reports of dead individuals were most frequent in May and August. Because the observations were collected unevenly, the results do not allow for the determination of population changes, mortality rates, or causes of death. However, they can assist authorities in planning seasonal and geographic monitoring, prioritizing the examination of dead porpoises, and coordinating conservation efforts. More precise information can support decision-making aimed at protecting these mammals and the natural heritage of the Black Sea.

## 1. Introduction

As key components of marine ecosystems, cetaceans make a significant contribution to the structure and functioning of marine food webs [1,2,3].

Occupying the upper levels of food chains, they are long-lived, highly mobile apex predators that integrate ecological processes across vast spatial and temporal scales and can respond to changes in prey availability, habitat conditions, and human activities. In this regard, their ecological importance extends beyond predator–prey interactions, as cetaceans can also influence nutrient cycling and ecosystem functioning [2]. Cetaceans are vulnerable to a range of anthropogenic pressures, including fishing and bycatch [4], underwater noise [5], maritime traffic [6], pollution [7,8], habitat degradation [9], and changes in prey availability [10].

Cetacean monitoring can provide essential information on species presence, group composition, and distribution, contributing to the identification of important habitats and the assessment of population trends when supported by standardized survey methods [11,12,13]. Furthermore, stranding data provide complementary insights into mortality patterns and causes, as well as potential threats to the species [14]. However, both types of observations have limitations. Monitoring live cetaceans depends directly on observation effort, the observer’s experience, detection probability, and environmental conditions [11,15,16,17], while stranding observations are influenced by the field effort dedicated to searching for and reporting cases, the accessibility of the location, the degree of carcass decomposition, their buoyancy, and environmental factors such as winds or currents [18,19,20]. All these factors must be considered when analyzing and interpreting spatial and temporal patterns, particularly in cases involving non-standardized observation efforts.

In the semi-enclosed basin of the Black Sea, characterized by specific environmental conditions and limited connectivity with the Mediterranean, the resident cetacean fauna comprises three taxa. Thus, due to the basin’s geographic isolation and unique environmental conditions, three regionally endemic subspecies have evolved: *Delphinus delphis ponticus* (the Black Sea common dolphin), *Tursiops truncatus ponticus* (the Black Sea bottlenose dolphin), and *Phocoena phocoena relicta* (the Black Sea harbor porpoise), which, although they share the same basin, are characterized by distinct ecological requirements and distribution patterns [21,22,23]. *D. d. ponticus* is generally associated with deeper waters, whereas *T. t. ponticus* shows a stronger association with shallower habitats. *P. p. relicta* also occupies a distinct habitat niche influenced by both depth and temperature [24]. Seasonal differences in occurrence frequency and group size have also been reported, indicating that the local presence and social structure of Black Sea cetaceans can vary considerably throughout the year [25,26].

According to IUCN assessments, *D. d. ponticus* is listed as vulnerable (VU), while *T. t. ponticus* and *P. p. relicta* are listed as endangered (EN) [27,28,29]. Although data regarding the abundance and distribution of cetaceans in the Black Sea were previously limited, this information gap has narrowed considerably over the last decade. The “ACCOBAMS Survey Initiative” and the CeNoBS project, which involved coordinated aerial monitoring across the waters of Bulgaria, Georgia, Romania, Turkey, and Ukraine, recorded 1744 sightings along approximately 7300 km of transects. Despite covering only 52% of the Black Sea basin, spatial model-based analyses of the surveyed area estimated populations of approximately 108,283 *D. d. ponticus*, 22,720 *T. t. ponticus*, and 93,808 *P. p. relicta*, totaling roughly 224,811 cetaceans [21]. These findings underscore the need for a continuous cetacean monitoring program at both local and regional levels to enhance species conservation efforts and more precisely identify the temporal and spatial patterns of these species.

More recently, the importance of continuous monitoring was also officially recognized in Romania through the National Action Plan for the Conservation of Black Sea Cetaceans, adopted in 2024 by the Ministry of Environment, Waters and Forests [30]. Within this plan, a national framework is established to improve the conservation of Black Sea cetaceans, and long-term monitoring is emphasized, including the assessment of cetacean populations, stranding incidents, and accidental bycatch, as well as post-mortem investigations and the identification of habitats important for the conservation of these marine mammals.

In accordance with the background information presented, the purpose of this study is to analyze data on cetaceans collected along the Romanian coast of the Black Sea during 2021–2025. By correlating sightings of live cetaceans with strandings data for the three taxa present in the Black Sea, this study characterizes the species composition and interannual variation in sightings, investigates seasonal patterns and group sizes, assesses distribution differences between coastal and offshore zones, and describes spatiotemporal patterns of recorded dead cetaceans. The study thus provides an updated assessment of cetacean presence along the Romanian area, information relevant to their long-term monitoring and conservation at both national and regional levels.

## 2. Materials and Methods

### 2.1. Opportunistic Cetacean Observations

Cetacean sighting data were collected between 2021 and 2025 in the Romanian sector of the Black Sea, primarily during scientific research cruises conducted for environmental monitoring and sample collection. Observations were made opportunistically during daylight hours, both while vessels were in transit and during sampling operations. Additional sightings were recorded from shore.

A total of 188 sighting events involving 1392 live individuals were included in the analysis, of which 153 events were recorded from vessels or other watercraft and 35 from shore. A sighting event was defined as the visual observation of one or more cetaceans at a given location and time. For each event, the species, number of individuals, and geographical location were recorded, together with the date. Species identification was based on visible morphological characteristics.

Observations were not conducted along predefined cetacean survey transects, and observation effort was not standardized among months or years. Although the dates, locations, and observation platforms were documented, information on periods without cetacean detections was unavailable. Consequently, total observation hours and distances travelled during active searching could not be quantified, preventing the calculation of standardized encounter rates. The dataset was therefore analyzed as opportunistic occurrence records rather than as a basis for estimating abundance or population trends. Given that the taxonomic composition of the dataset could also have been influenced by interannual variation in the proportion of observations made from vessels versus from the shore, comparisons between years were strictly descriptive in nature. They were limited to the available records, and the observed differences were not interpreted as evidence of changes in abundance, population composition, or distribution.

### 2.2. Cetacean Stranding Data

Records of dead cetaceans were collected between 2021 and 2025 along the Romanian Black Sea coast and in adjacent marine waters. Observations were made directly by the authors participating in scientific expeditions during the study period. Field observations were conducted both along the coastline and aboard research vessels. A total of 76 dead cetaceans were documented.

For each record, the available information included the date, geographical location, species identification, and number of individuals. Carcasses were photographed, and biometric measurements were taken whenever their condition allowed. As individuals were found at different stages of decomposition, reliable species identification and biometric measurements were not always possible. Species identification was based on visible morphological characteristics, while individuals that could not be reliably identified were classified as unidentified cetaceans.

No necropsies were performed as part of this study, and causes of death could not be determined. Furthermore, because observations were opportunistic and detection effort was not standardized, the dataset was used to describe the taxonomic composition and spatial and temporal distribution of recorded dead cetaceans, rather than to estimate mortality rates.

### 2.3. Statistical Analysis of Data

All statistical analyses were performed in RStudio software (version 2026.07.1+147; Posit Software, PBC, Boston, MA, USA), with statistical significance set at *p* < 0.05. Live cetacean records were summarized by species and year using the number of individuals, sighting events, and relative frequencies (%). A sighting event was defined as an encounter with a cetacean individual or group. Successive observations of the same group during continuous visual tracking were treated as a single event to avoid double counting. Photo-identification was not performed; therefore, repeated encounters with the same individuals or groups could not be entirely excluded. Individual counts and sighting events were considered separately to account for variation in group size. Group size was summarized using the median, interquartile range (IQR), and minimum–maximum values, and species-specific coastal–offshore distributions were assessed using the minimum distance from each live sighting to the coastline. Differences in group size among species and years were assessed using Kruskal–Wallis tests. Significant results were followed by Dunn’s post hoc tests with Holm correction, and effect size was expressed as epsilon-squared (ε^2^).

Seasonal patterns were analyzed using live sighting events as independent observational units. Differences in monthly distributions among species were evaluated using Pearson’s chi-square test of independence. Because of low expected cell frequencies, *p*-values were estimated using Monte Carlo simulation with 200,000 replicates (chisq.test, simulate.*p*.value = TRUE, B = 200,000). Association strength was quantified using Cramér’s V, and standardized Pearson residuals were examined following a significant overall test to identify species–month combinations contributing most strongly to the association.

Records of dead cetaceans were summarized by species, year, and month using absolute counts and relative frequencies. Unidentified individuals were included in overall mortality totals but excluded from species-level comparisons. Differences in monthly distributions of recorded dead cetaceans among species were evaluated using Pearson’s chi-square test with Monte Carlo *p*-values based on 200,000 replicates. Spatial differences in dead cetacean records among species were assessed using latitudinal coordinates and the Kruskal–Wallis test. Finally, species-specific dead-to-live ratios were calculated descriptively as the number of identified dead individuals per 100 live individuals recorded; these values were used only as descriptive indices and were not interpreted as mortality rates because observation effort was not standardized.

The minimum distance from each live cetacean sighting to the coastline was calculated using the GSHHG coastline dataset (intermediate resolution). Sighting coordinates and coastline geometries were projected to WGS 84/UTM zone 35N (EPSG:32635), and the shortest Euclidean distance between each observation point and the nearest coastline segment was calculated and expressed in kilometers.

Sighting locations were mapped using ArcMap 10.6.1 (Esri, Redlands, CA, USA) to describe the spatial distribution of recorded cetacean observations within the Romanian Black Sea sector. Spatial concentrations were analyzed using Kernel Density Estimation (KDE), considering both the location of each sighting event and the number of cetacean individuals recorded. The resulting density surface was used to visualize areas with different concentrations of recorded individuals.

## 3. Results

### 3.1. Live Cetacean Sightings

In total, 1392 cetaceans were recorded during 188 sighting events between 2021 and 2025 in the Romanian Black Sea sector. Regarding the breakdown by species, *T. t. ponticus* accounted for 44.7% (*n* = 622) of the observed individuals, followed by *D. d. ponticus* (43.6%; *n* = 607) and *P. p. relicta* (11.7%; *n* = 163) (Figure 1).

#### 3.1.1. Composition and Interannual Variation in Live Cetacean Records

The composition of recorded live cetacean sightings varied among years (Figure 2). In 2021, *D. d. ponticus* and *T. t. ponticus* contributed similarly to the total number of live individuals (46.7% and 45.7%, respectively), whereas *D. d. ponticus* represented 66.9% of individuals recorded in 2023. *T. t. ponticus* accounted for 57.4% of recorded individuals in 2024 and 92.4% in 2025. No *D. d. ponticus* sightings were recorded in 2025. These proportions describe the available records only; differences in observation opportunities, platforms, and spatial coverage prevent population-level interpretation.

Differences were also observed in the number of sighting events, as *D. d. ponticus* accounted for 49 out of 87 live sighting events in 2021 and 17 out of 27 events in 2023, whereas *T. t. ponticus* accounted for 19 out of 22 events in 2025. As the observation effort was not standardized among years, the observed differences describe variations in the recorded sightings and therefore should not be interpreted as population trends. Observation effort and the relative contribution of vessel-based and shore-based records varied among years. Therefore, these results describe interannual differences in the composition of recorded sightings and should not be interpreted as changes in population abundance or species distribution.

#### 3.1.2. Monthly Distribution of Recorded Sighting Events

The monthly distribution of recorded sighting events differed significantly among the three subspecies (Pearson’s χ^2^ = 50.02, Monte Carlo *p* < 0.001; Cramér’s V = 0.365). *D. d. ponticus* was recorded more frequently than expected in September (standardized residual, SR = 3.28) and less frequently in August (SR = −2.13). In contrast, *T. t. ponticus* was recorded more frequently than expected in August (SR = 3.32), March (SR = 2.65), October (SR = 2.55), and November (SR = 2.29), and less frequently in September (SR = −4.81). No individual month showed a standardized residual exceeding ±1.96 for *P. p. relicta*. The association was primarily associated with contrasting monthly patterns in the recorded sightings of *D. d. ponticus* and *T. t. ponticus*, particularly during August and September.

#### 3.1.3. Coastal-Offshore Distribution of Live Cetacean Records

The distance of recorded live cetacean sightings from the coastline differed significantly among the three subspecies (Kruskal–Wallis: H = 68.42, df = 2, *p* < 0.001; ε^2^ = 0.359). *T. t. ponticus* were recorded closest to the coastline, with a median distance of 4.40 km (interquartile range; IQR: 1.53–22.88 km). *D. d. ponticus* sightings occurred farther offshore, with a median distance of 61.78 km (IQR: 30.71–124.75 km). *P. p. relicta* sightings showed an intermediate median distance of 30.44 km (IQR: 4.69–113.73 km) (Figure 3).

The Dunn’s post hoc tests with Holm correction indicated significant differences between all species pairs: *D. d. ponticus* vs. *P. p. relicta* (adjusted *p* = 0.0067), *D. d. ponticus* vs. *T. t. ponticus* (adjusted *p* < 0.001), and *P. p. relicta* vs. *T. t. ponticus* (adjusted *p* = 0.0003). These results describe differences in the distance of recorded sightings from the coastline rather than species-specific habitat preferences.

#### 3.1.4. Species-Specific Variation in Group Size

The group size varied considerably within the species. Groups of *D. d. ponticus* and *T. t. ponticus* ranged from 1 to 100 individuals, whereas those of *P. p. relicta* ranged from 1 to 40 individuals. Median group size was 4 individuals for *D. d. ponticus* (interquartile range; IQR: 2–6) and *T. t. ponticus* (IQR: 2–10), and 2.5 individuals for *P. p. relicta* (IQR: 2–5). Despite these differences, group size did not differ significantly among the three species (Kruskal–Wallis: H = 2.95, df = 2, *p* = 0.229).

When records from all three species were pooled, the group size varied significantly among years (Kruskal–Wallis: H = 18.38, df = 4, *p* = 0.001; ε^2^ = 0.079). The median group size ranged from 2 individuals in 2022 (IQR: 1–4) to 5 individuals in 2024 (IQR: 3–7.5). The Dunn’s post hoc tests with Holm correction indicated that groups recorded in 2022 were significantly smaller than those recorded in 2021 (adjusted *p* = 0.0065), 2023 (adjusted *p* = 0.0278), and 2024 (adjusted *p* = 0.0300). No significant differences were found between the remaining pairs of years.

### 3.2. Stranded Cetaceans

During 2021–2025, 76 stranded cetaceans were recorded along the Romanian Black Sea coast, of which 58 (76.3%) were identified to species level. *P. p. relicta* accounted for most identified individuals (*n* = 41; 70.7%), followed by *T. t. ponticus* (*n* = 14; 24.1%) and *D. d. ponticus* (*n* = 3; 5.2%). The remaining 18 individuals were unidentified; 17 of them, recorded in 2022, were found in an advanced state of decomposition, consisting of skeletal remains that precluded species identification (Figure 4).

#### Composition and Interannual Variation in Stranded Cetaceans’ Records

The number of stranded cetaceans varied over the years, from 4 individuals in 2021 to 24 in 2022 and a maximum of 25 in 2023, followed by 15 in 2024 and 8 in 2025. *P. p. relicta* became particularly prominent during 2023–2025, representing 66.7% of identified deaths in 2023, 80.0% in 2024, and 87.5% in 2025. Across these three years, the species accounted for 35 of 47 (74.5%) identified dead cetaceans.

The number of recorded dead cetaceans varied among months, with the highest number of dead individuals recorded in August (*n* = 27; 35.5%), followed by May (*n* = 15; 19.7%). Together, these two months accounted for 55.3% of all recorded dead individuals. Among the 58 individuals identified to species level, *P. p. relicta* showed the highest number of recorded dead individuals in August (*n* = 12), followed by May (*n* = 7), April (*n* = 6), and September (*n* = 5). However, the monthly distribution of mortality did not differ significantly among species (Pearson’s χ^2^ = 20.27, Monte Carlo *p* = 0.42).

### 3.3. Descriptive Comparison of Live Records and Recorded Mortality

The comparison of live cetacean sightings and dead-animal records revealed differences in the representation of the three subspecies. During the study period, 607 live and 3 dead individuals of *D. d. ponticus*, 622 live and 14 dead individuals of *T. t. ponticus*, and 163 live and 41 dead individuals of *P. p. relicta* were recorded. These values corresponded to descriptive ratios of 0.49, 2.25, and 25.15 recorded dead individuals per 100 live individuals, respectively. However, although both datasets covered the same geographical region and study period, they were collected through different observation processes, without standardized sampling effort. Consequently, these ratios describe only the relative representation of each subspecies within the two datasets and cannot be interpreted as mortality rates or indicators of species-specific mortality risk. Notably, *P. p. relicta* accounted for the largest proportion of identified dead cetaceans (70.7%), despite representing only 11.7% of recorded live individuals.

## 4. Discussion

This study provides a multi-annual (2021–2025) assessment of the spatio-temporal distribution and mortality, within the Romanian sector, of the three subspecies of cetaceans present in the Black Sea: *T. t. ponticus*, *P. p. relicta* and *D. d. ponticus*. The pattern of cetacean sightings varied notably between 2021 and 2025. Overall, among the three observed species, *T. t. ponticus* (44.7%) and *D. d. ponticus* (43.6%) accounted for similar proportions of the total number of individuals observed, whereas sightings of *P. p. relicta* (11.7%) were less frequent. Also, the species representation varied from year to year, with *D. d. ponticus* showing higher frequencies in 2021 and 2023, and *T. t. ponticus* being predominant in 2024 and, especially, in 2025.

Our observations align with the variations reported within other studies from across the entire Black Sea. All three species were observed in the Romanian sector of the Black Sea, with densities varying by season. The *P. p. relicta* showed lower abundances in spring (359 individuals) compared to summer, when up to 5697 individuals were observed. The *T. t. ponticus* abundance decreased from 667 individuals in spring to 451 individuals in summer, while the *D. d. ponticus* was observed (163 individuals) only in summer [31].

On a broader scale, the heterogeneous spatial distribution of Black Sea cetaceans was also highlighted within the ASI/CeNoBS aerial survey conducted in the summer of 2019, when cetaceans’ abundance in the surveyed area was estimated at approximately 108,283 *D. d. ponticus*, 93,808 *P. p. relicta*, and 22,720 *T. t. ponticus*. Higher densities, up to 0.697 individuals/km^2^, of common dolphins were seen in the south of the basin, mostly in the Turkish part, compared to the Romanian waters (0.130 individuals/km^2^). Conversely, the Romanian waters were found to be a regional hotspot for *P. p. relicta*, exhibiting densities up to 0.995 individuals/km^2^, compared to, for example, the Bulgarian sector where the density reached only 0.337 individuals/km^2^. The *T. t. ponticus* densities varied from approximately 0.194 to 0.195 individuals/km^2^ in the Romanian and Bulgarian sectors [21]. *T. t. ponticus* (*n* = 618) was also the most abundant species, followed by *D. d. ponticus* (*n* = 554), following opportunistic observations between 2006–2013 in the Bulgarian waters, as reported by [32]. In the north-western part of the basin, in the shallower waters, *P. p. relicta* were by far the most abundant observed species, with abundances of 175 individuals and densities of 1.51 individuals/km^2^, compared to *T. t. ponticus* (*n* = 31; 0.22 individuals/km^2^) and *D. d. ponticus* (*n* = 59; 0.51 individuals/km^2^) [33].

The differences between various regions of the Black Sea basin illustrate the variation in the three species depending on the observation periods and the specific areas selected. Consequently, the recorded annual variations should be viewed and interpreted as fluctuations in presence rather than as population trends. Factors influencing the annual totals include the frequency of observations, the occasional occurrence of large groups of individuals, and uneven observation effort. Thus, the data from this study provide a recent description of recent cetacean occurrence along the Romanian coast.

The taxonomic composition of recorded sighting events differed among months. The most pronounced contrast was noted between *D. d. ponticus* and *T. t. ponticus*, with the former being disproportionately associated with the month of September, whereas *T. t. ponticus* was most frequently sighted in August and very rarely in September. Regarding *P. p. relicta*, no significant associations were observed between the month of sighting and the species.

Such seasonal variations have also been reported in other studies within the Black Sea basin, with differences regarding the season in which the highest number of individuals was recorded. For the southwestern sector of the basin, Uluduz et al. [26] report peak values in April–May. Similarly, for the southern part, the highest numbers of specimens are observed in spring [34]. However, the monitored area differed between the seasons. Popov et al. [35] reported higher numbers of *P. p. relicta* offshore over the southern continental shelf during autumn, and fewer near the coast than in the summer.

Previous studies have linked the distribution of marine mammals to sea surface temperatures and water depth [24], while prey movement and availability have been considered potential factors influencing cetacean distribution and temporal variation [26]. However, in the absence of data regarding environmental conditions and prey, testing these relationships was not possible within the scope of this study. On a monthly basis, variations in the taxonomic composition of recorded observations may reflect the combined effect of biological differences, unequal observation effort, spatial coverage, and detectability. Associating these variations with specific ecological factors or interpreting them as seasonal changes in the number of recorded individuals is therefore not justified.

Regarding the present study, given the lack of available data on environmental factors and prey species for the period under review, these aspects cannot be correlated or validated. On the other hand, the data analyzed highlight the need for multi-seasonal cetacean monitoring and caution against extrapolating local seasonal patterns to the entire Black Sea region.

A gradient in the distribution of the three species from the coast towards the open sea was also observed, with *T. t. ponticus* found closest to shore (median value: 4.40 km), followed by *P. p. relicta* (30.44 km) and *D. d. ponticus* (61.78 km) at the greatest distance, consistent with other studies in the Black Sea and also with the ecology of the three species [36]. The observations made in the Romanian sector of the Black Sea show that the species *T. t. ponticus* and *D. d. ponticus* were recorded at different distances from the coastline, although uneven survey coverage prevents inference of distinct habitat boundaries. However, the distance from the shore does not equate to water depth, so the results obtained do not necessarily indicate a bathymetric preference. For future studies, bathymetric data and environmental variations would allow for a direct assessment of these habitat relationships.

Although there was considerable variation within each species, group size did not differ significantly between species. For *T. t. ponticus* and *D. d. ponticus*, the median group size was 4 individuals, while for *P. p. relicta* it was 2.5 individuals, although much larger groups were sometimes observed.

Similar values were also reported by Uludüz et al. [26], who observed median group sizes of three individuals for *D. d. ponticus*, two for *T. t. ponticus,* and one for *P. p. relicta* during 29 months of monitoring in Zonguldak, in the southwestern part of the basin. In the southern area off the Bulgarian coast, although the observations do not specify median group sizes for each species, small groups of *T. t. ponticus* were frequently reported near fishing vessels, particularly during trawl hauling [32]. It should also be noted that comparisons with other studies must be made cautiously, considering the nature of the observations, as Uludüz et al. [26] conducted observations from the shore, whereas the data from the Bulgarian area were collected opportunistically from various vessels [32]. Therefore, when observations spanning multiple years and seasons are pooled, temporal variations and occasional large-scale aggregations can obscure interspecific differences; consequently, the absence of an overall difference between species does not imply identical grouping patterns.

Among the three monitored species, *P. p. relicta* was predominant in terms of strandings, accounting for 70.7% of the specimens identified to the species level. In the western Black Sea basin, Popov & Meshkova [14] report similar figures for the Bulgarian sector during the 2020–2022 period. Likewise, regarding the Turkish coast, Tonay et al. [37] found that approximately 48% of specimens stranded between 2007 and 2009 were *P. p. relicta* individuals. Temporal variations in stranding data are also evident in this case. For instance, Tonay [38] reports *T. t. ponticus* as the dominant species during the 2012–2013 period.

The end of spring and summer, specifically May and August, saw the highest number of stranding cases recorded along the Romanian coast and in neighboring regions. Most *P. p. relicta* and *T. t. ponticus* carcasses along the Bulgarian coast were observed during July and August [14]. Regarding the Turkish coast, 50 stranded cetaceans were recorded between the summer of 2007 and the spring of 2009, with the highest stranding rate (0.3 individuals/km) reported in the summer of 2007. Furthermore, 83% of *P. p. relicta* were found stranded during spring and summer, with no cases recorded in winter [37].

Although when analyzed collectively, observations of cetacean strandings may present a recurring pattern of carcass recovery in the western Black Sea, the detection of carcasses depends on cetacean distribution, decomposition, drift, coastal currents, monitoring effort, and the probability of reporting. Thus, strandings should not be interpreted as a direct measure of the timing or magnitude of mortality.

In the context of mortality associated with fishing activities in the Black Sea, the high proportion of the subspecies *P. p. relicta* among the recorded dead cetaceans is particularly significant: 41 out of the 58 identified specimens (70.7%). Popov et al. [39] identified incidental capture in fishing gear, particularly bottom-set gillnets targeting turbot, as a major threat to the Black Sea harbour porpoise. During monitored fishing operations, they recorded 182 accidentally caught *P. p. relicta*, compared with four *T. t. ponticus* and three *D. d. ponticus*, with the highest bycatch risk occurring between April and July. They estimated an annual bycatch of approximately 11,826–16,200 harbour porpoises across the Black Sea. Romanian stranding records from 2019–2020 likewise showed a high representation of this subspecies. Consequently, intensifying the monitoring of strandings during these months, in coordination with the monitoring of fishing activities, could be considered a priority, even though these specific months cannot be classified as periods with a proven risk of incidental catches (bycatch). Given that no necropsies were performed as part of this study, it is not possible to attribute recorded deaths to interactions with fishing activities.

Our interpretations are also consistent with ACCOBAMS Resolution 9.14, which identifies incidental catch as the primary source of human-induced mortality for the Black Sea harbour porpoise. The Resolution encourages monitoring via independent observers and, whenever feasible, the recovery of incidentally caught dead specimens for the purpose of conducting necropsies [40]. Establishing a link between the strandings recorded in Romania and interactions with fisheries would require standardized carcass surveys, with documented search effort, as well as prompt post-mortem examinations. This should be supplemented by data on fishing effort, the characteristics of the gear used, and independently recorded incidental catches, all correlated in terms of time and space. Furthermore, assessing the relationship between the locations where carcasses were recovered and the areas where the deaths may have occurred would require information regarding carcass drift.

Furthermore, given that the location where a stranded cetacean is found may differ from the place of death, the sites where stranded cetaceans are discovered must be interpreted with caution, as the absence of significant species-specific north–south differences does not imply similar mortality distributions. Factors such as waves, wind, currents, decomposition, scavengers, area accessibility, and reporting effort can influence carcass recovery. For all the dead cetaceans documented in this study, the causes of death remained undetermined, and the lack of systematic post-mortem investigations constitutes a major limitation. By incorporating standardized external examinations, biometric measurements, necropsies, histopathological analyses, and appropriate tissue sampling, future monitoring of strandings could make a more significant contribution to conservation. Assessing the potential causes of death, health status, and demographic characteristics of the affected animals would be enhanced by correlating these investigations with fisheries monitoring and data on carcass drift.

Regarding the differences between the number of live sightings and strandings, the species *P. p. relicta* showed the greatest contrast, accounting for 11.7% of all recorded live sightings compared to 70.7% of all identified dead specimens. These results translate to 25.15 dead individuals per 100 recorded live sightings of *P. p. relicta*, versus 2.25 for *T. t. ponticus* and 0.49 for *D. d. ponticus*. The high proportion of *P. p. relicta* in the mortality data is consistent with regional data on strandings and bycatch [35,37,39].

Given that different protocols are used for observing live and stranded cetaceans, the comparison serves only to highlight the disproportionate representation of the species *P. p. relicta* in the dead cetaceans dataset. However, despite this limitation, the observed pattern is highly relevant for conservation monitoring purposes. The results presented in this study, alongside other evidence from the Black Sea basin regarding bycatch, underscore the need to pay special attention to *P. p. relicta* through systematic mortality monitoring, the performance of necropsies, and the monitoring of interactions with fishing activities.

By accumulating local records over several years, this dataset contributes to conservation efforts, complementing standardized population assessments. To ensure adequate sampling of all three taxa, future monitoring should encompass both coastal and offshore waters, as suggested by the differences in the locations of the observations. Furthermore, studies with balanced seasonal coverage would allow for the testing of hypotheses derived from monthly variations in the taxonomic composition of the observations. However, it is not possible to determine habitat preferences, periods of peak seasonal abundance, or population trends based on these patterns, as observation effort and detection probability have not been quantified. Instead, their value lies in guiding the spatial and temporal planning of future standardized studies.

Regarding the implementation of Romania’s National Action Plan for the Conservation of Black Sea Cetaceans [30], stranding records highlight several practical needs. The necessity for early detection, rapid reporting, and prompt examination is underscored by the 18 carcasses that remained unidentified due to advanced decomposition, 17 of which were recorded in 2022. This degradation demonstrates how the condition of a carcass can lead to the loss of taxonomic information. Additionally, the investigation of porpoise strandings warrants priority attention, given the predominance of *P. p. relicta* individuals among the identified carcasses. However, the causes of death and any potential link between mortality and accidental bycatch cannot be determined from these records, as no necropsies were performed. Investigating mortality potentially related to fishing would benefit from correlating standardized stranding records and post-mortem findings with independent data on fishing activities and accidental bycatch. Within the national conservation framework, these findings can thus support the planning of coordinated monitoring and intervention activities in the event of strandings. To distinguish variations in carcass recovery from those associated with survey coverage, it is necessary to document the search effort for each sector of the Romanian coast included in the study. Based on current records, no areas with high mortality linked to fishing activities can be identified.

## 5. Conclusions

The 2021–2025 observations provide an updated characterization of cetacean occurrence in the Romanian Black Sea, based on 1392 individuals recorded during 188 live sighting events. *T. t. ponticus* and *D. d. ponticus* were similarly represented, whereas *P. p. relicta* accounted for a considerably smaller proportion of live records.

Recorded sighting locations differed significantly among species along the coastal-offshore gradient. *T. t. ponticus* occurred predominantly closer to shore (median 4.40 km), *P. p. relicta* showed an intermediate distribution (30.44 km), and *D. d. ponticus* was recorded considerably farther offshore (61.78 km), with significant differences among all species pairs.

The monthly composition of recorded sightings differed, with the clearest differentiation between *D. d. ponticus*, disproportionately recorded in September, and *T. t. ponticus*, more frequently observed in August. However, interpreting these differences as abundance-related seasonal variations or explaining them through ecological factors is not possible because of unequal observation effort, spatial coverage and unquantified detectability. But, through standardized monitoring in different seasons, hypotheses formulated on these observations may be tested.

Dead cetacean records showed a distinct pattern of carcass recovery: 76 dead cetaceans were documented during 2021–2025, with *P. p. relicta* accounting for 70.7% of individuals identified to species level. More than half of all recorded dead individuals occurred in August and May, although these records reflect carcass recovery patterns rather than direct estimates of mortality.

By documenting the presence of cetaceans and the recovery of dead specimens in Romanian waters during the 2021–2025 period, these opportunistic records complement existing population assessments. They provide practical benchmarks for planning multi-seasonal monitoring in both coastal and offshore areas and highlight the need for the prompt examination of dead specimens, as well as the coordination of investigations in cases of strandings. The implementation of Romania’s National Action Plan could be supported by integrating these activities with standardized population assessments and fisheries monitoring. However, it is not possible to determine population trends, mortality rates, or causes of death based on the data presented.

## Figures and Tables

**Figure 1:**
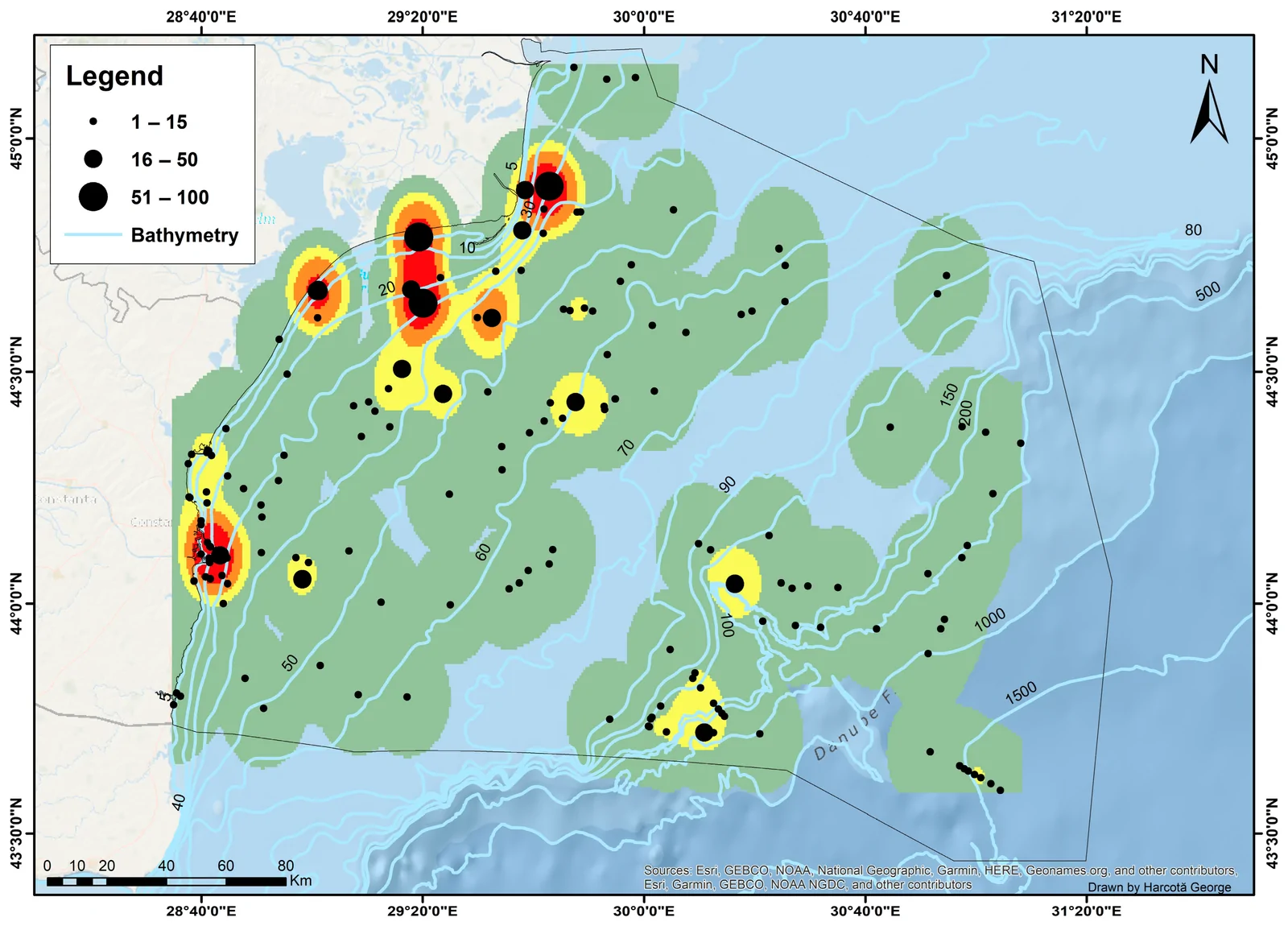
Spatial distribution of live cetacean sightings in the Romanian sector of the Black Sea during 2021–2025. Black circles represent individual sighting events. The black circles represent individual sighting events, with symbol size proportional to the number of cetaceans recorded per event: 1–15 individuals, 16–50 individuals, and 51–100 individuals. The coloured areas represent a spatial concentration surface derived from recorded observations, ranging from lower concentrations (green) to higher concentrations (red), with intermediate values shown in yellow. Light blue lines represent bathymetric contours, labelled with water depth in metres.

**Figure 2:**
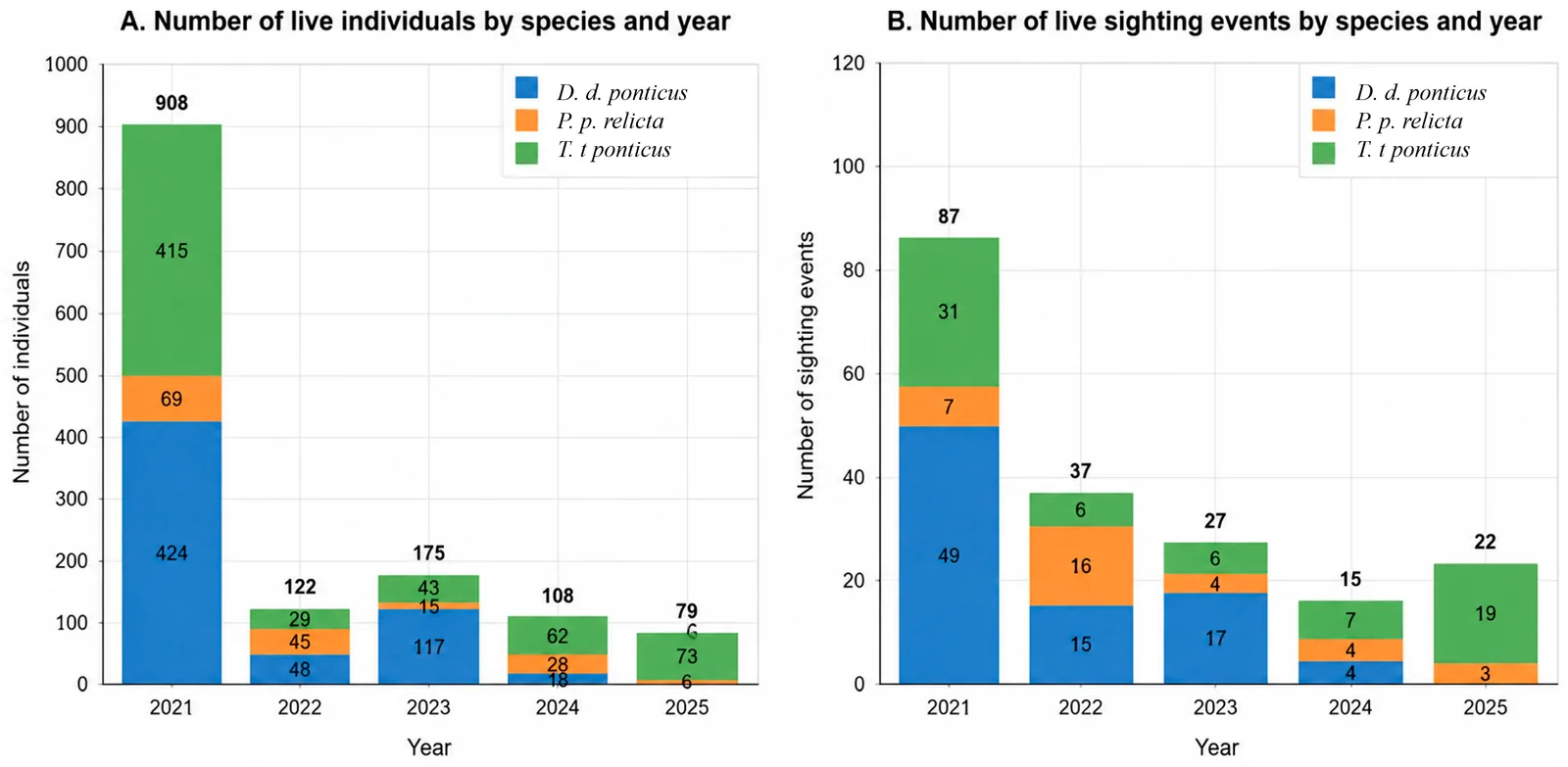
Annual numbers of recorded live cetaceans (**A**) and sighting events (**B**) by subspecies in the Romanian Black Sea sector (2021–2025). Values indicate subspecies-specific counts and annual totals. Data represent opportunistic observations with non-standardized sampling effort and should not be interpreted as population trends.

**Figure 3:**
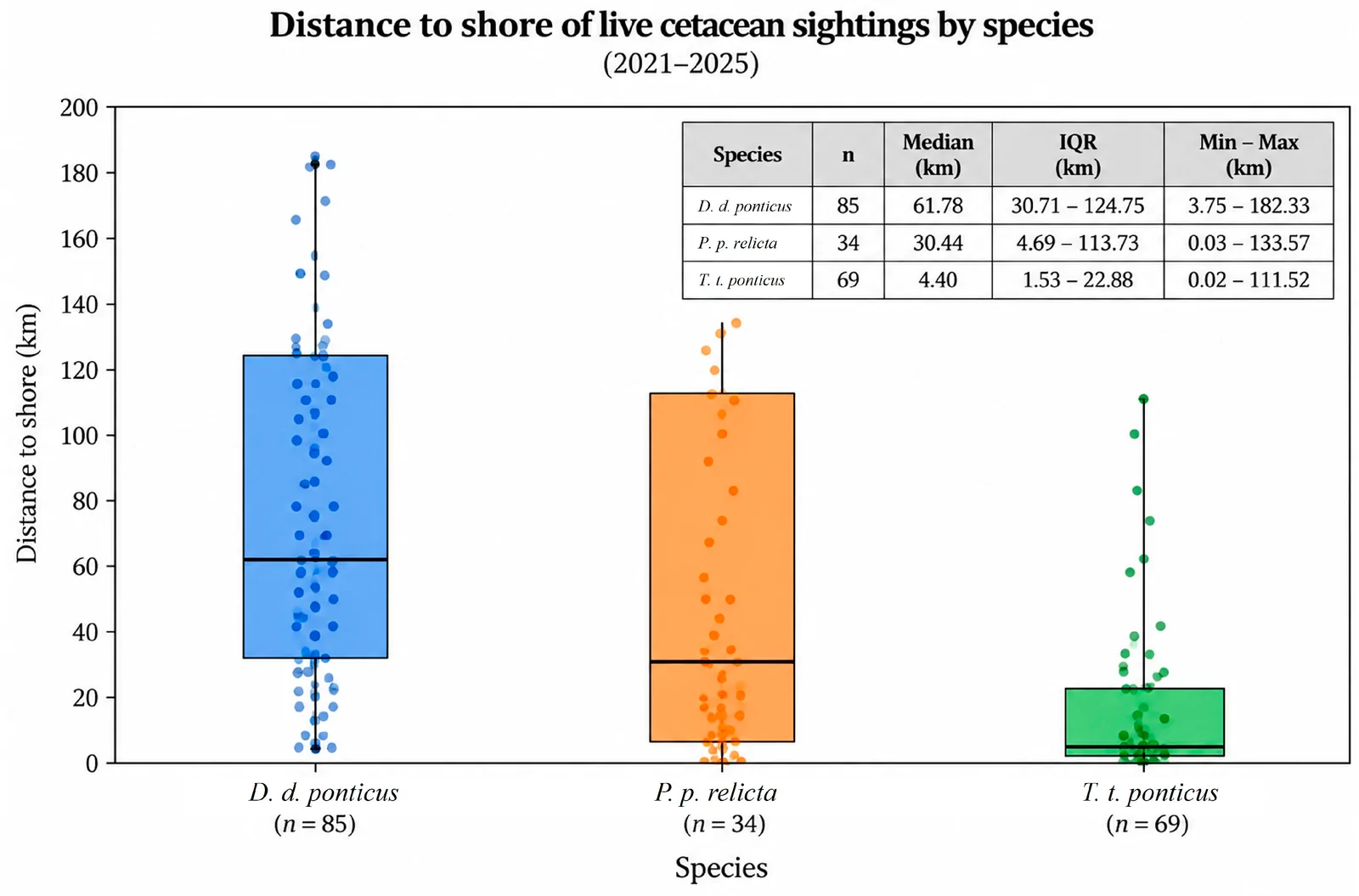
The distance to shore (km) of live cetacean sightings by species between 2021 and 2025.

**Figure 4:**
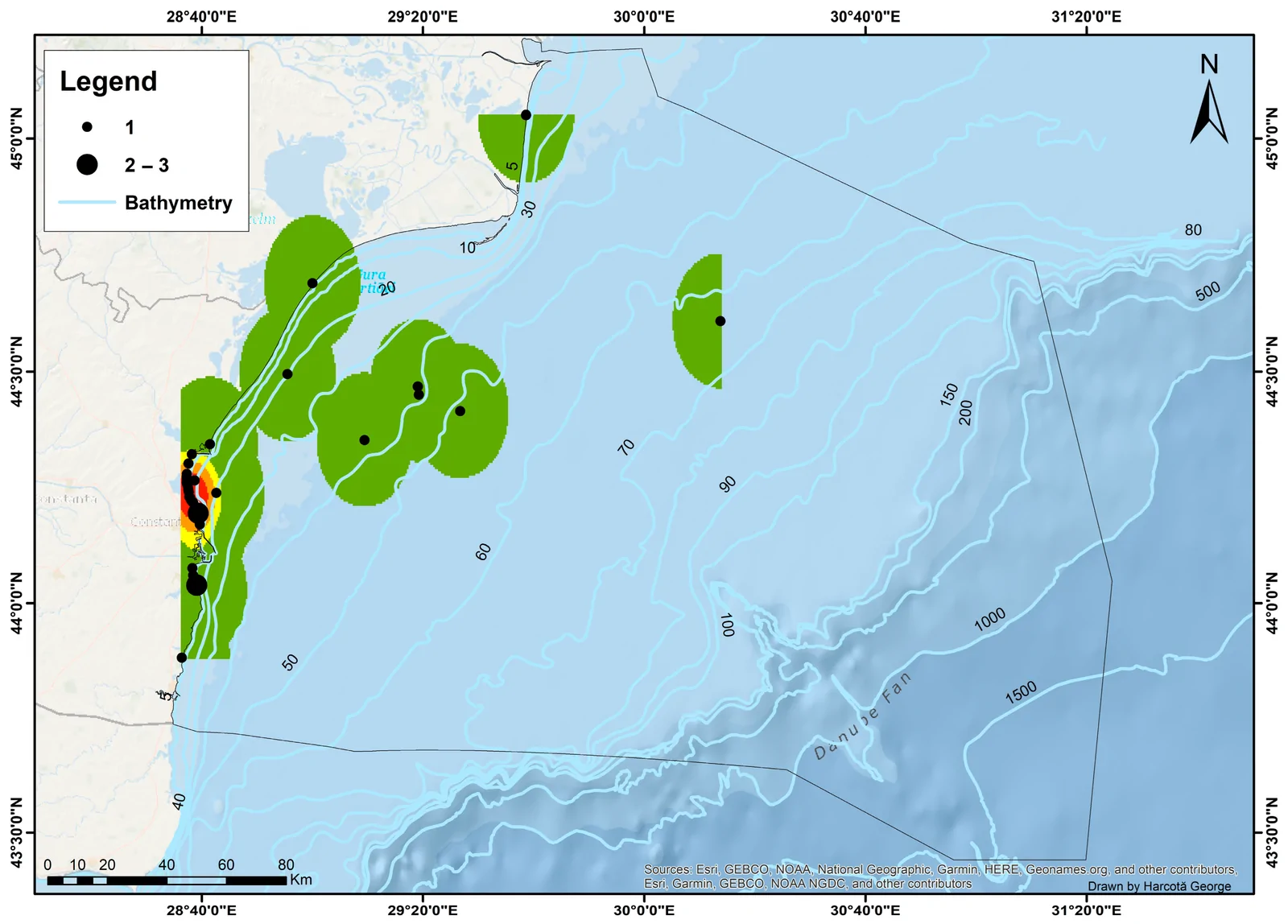
The spatial distribution and aggregation of stranded cetacean sightings in the Romanian sector of the Black Sea during 2021–2025. The black circles represent individual sighting events, with symbol size proportional to the number of cetaceans recorded per event (1 and 2–3 individuals). The coloured areas represent a spatial concentration surface derived from recorded observations, ranging from lower concentrations (green) to higher concentrations (red), with intermediate values shown in yellow. Light blue lines represent bathymetric contours, labelled with water depth in metres.

## Data Availability

The data presented in this study are available on request from the corresponding author, as the research is still ongoing and the data are subject to internal restrictions.
